# Ergochromes: Heretofore Neglected Side of Ergot Toxicity

**DOI:** 10.3390/toxins11080439

**Published:** 2019-07-25

**Authors:** Miroslav Flieger, Eva Stodůlková, Stephen A. Wyka, Jan Černý, Valéria Grobárová, Kamila Píchová, Petr Novák, Petr Man, Marek Kuzma, Ladislav Cvak, Kirk D. Broders, Miroslav Kolařík

**Affiliations:** 1 Laboratory of Fungal Genetics and Metabolism, Institute of Microbiology of the Czech Academy of Sciences, Vídeňská 1083, CZ-14220 Prague, Czech Republic; 2 Department of Bioagricultural Sciences and Pest Management, Colorado State University, Fort Collins, CO 80523, USA; 3 Department of Cell Biology, Faculty of Science, Charles University, Viničná 7, CZ-128 00 Prague, Czech Republic; 4 TEVA Czech Ind, CZ-74770 Opava, Czech Republic

**Keywords:** mycotoxins, ergot alkaloids, ergochromes, secalonic acid, food safety, cereals, tetrahydroxanthones, *Claviceps*

## Abstract

Ergot, fungal genus *Claviceps*, are worldwide distributed grass pathogens known for their production of toxic ergot alkaloids (EAs) and the great agricultural impact they have on both cereal crop and farm animal production. EAs are traditionally considered as the only factor responsible for ergot toxicity. Using broad sampling covering 13 ergot species infecting wild or agricultural grasses (including cereals) across Europe, USA, New Zealand, and South Africa we showed that the content of ergochrome pigments were comparable to the content of EAs in sclerotia. While secalonic acids A–C (SAs), the main ergot ergochromes (ECs), are well known toxins, our study is the first to address the question about their contribution to overall ergot toxicity. Based on our and published data, the importance of SAs in acute intoxication seems to be negligible, but the effect of chronic exposure needs to be evaluated. Nevertheless, they have biological activities at doses corresponding to quantities found in natural conditions. Our study highlights the need for a re-evaluation of ergot toxicity mechanisms and further studies of SAs’ impact on livestock production and food safety.

## 1. Introduction

Ergot, the genus *Claviceps* (Ascomycota: Hypocreales) includes obligate plant parasitic fungi that develop in the ovary of grasses (including cereals), sedges, and rushes and form sclerotia containing toxins. The famous rye ergot, *Claviceps purpurea*, is a member of the section *Claviceps* which is specified by the production of highly toxic ergopeptines [1]. Recently it has been shown, that *C. purpurea sensu lato* (*s. l.*) is a complex of four cryptic species with different host grass spectra. While common land grasses are often infected by both *C. purpura sensu stricto* (*s. s.*) and *C. humidiphila*, cereal crops seem to be infected by just *C. purpurea s. s.* In the Palearctic region, these two species are the most important from an agricultural point of view [2,3]. Furthermore, recent findings suggest further cryptic diversification among North American *C. purpurea s. l.* specimens and their impacts on agricultural remains uncertain [4].

Ergot poisoning causes ergotism in humans and livestock. Most of the research related with ergotism has been focused on the ergot alkaloids (EAs) as these are among the most important natural pharmaceuticals and toxins in human history [5,6]. There is still some pharmaceutical research being conducted on EAs, however, research into their toxic effects on human health have relatively diminished. Humans are no longer at risk of ergotism in most of the world due to advanced seed cleaning and food screening for the presence of EAs. However, there has been a resurgence of research interested in the toxicoses of livestock or wild animals in recent years which has brought to light the substantial challenge of elucidating alkaloid-induced effects of animal responses to exposure [7,8]. Currently, the EU Scientific Panel on Contaminants in the Food Chain of the European Food Safety Authority (EFSA) recommended 12 priority alkaloids for monitoring in food and feed, all of which are grouped in the ergopeptines produced by *C. purpurea s. l*.

A recent review by Klotz [9] detailed the collective knowledge on ergotism and fescue toxicoses of livestock. He noted the complexity of this area of research as EA toxicity is affected by changes in alkaloid concentrations, proportions, and availability as well as individual’s genetic predispositions, prior exposures, and ambient environments. This led to the overall conclusion that the impacts of EAs on livestock, especially convulsive (neurological and abortogenic) symptoms are not caused by the sole action of a single toxin, but rather the combined impact and synergistic action of multiple EAs derived from *Claviceps* and *Epichloë* species [9,10]. While most of the available data of EAs effects on animals both address the symptoms and define the problem, there is still a lack of research on other fungal metabolites and their potential harmful effects on livestock and humans.

In *C. purpurea s. l.*, the average EAs content in sclerotia varies between studies and ranges from 0.01–1.3 mg/g [11,12,13,14,15,16] to 2.88–7.26 mg/g [17], with individual values rarely reaching 5–10 mg/g (d/w) [12,16,18]. In addition to EAs, *C. purpurea* produces many other secondary metabolites. Most have been identified and inspected for toxicity while others have still eluded proper examination, with no research on their effects on livestock [10]. In addition, some of these other metabolites are produced in greater quantity than the heavily researched EAs. Ergot sclerotia can contain 1–2% of pigments, predominantly yellow biphenyl pigments called ergochromes (ECs) [19], which typically reach 5 mg/g of the sclerotia dry weight [20]. The main ECs of ergot are secalonic acids A–C (SAA, SAB, SAC), whereas related ergoflavin, ergochrysin A, B and chrysergonic acid are produced in negligible amounts. Other minor pigments, such as anthraquinone derivates endocrocin and clavorubin, are present [21]. Secalonic acids D–F were described from various moulds and lichens [22,23]. Secalonic acids exhibit various biological activities with the best studied secalonic acid D (SAD) showing mutagenic, teratogenetic, and cytotoxic activity [24,25,26]. Strong biotoxic activity against animals, plants, or microbial cells was also documented in secalonic acid A [27,28], F [29], and G [30]. 

Surprisingly, across the distribution of ergot species, the quantity and environmental role of ECs are still unknown, despite their proved activity against mammalian cells. There is currently no knowledge on the effect of ECs on livestock or how synergistic actions of ECs with other ECs or EAs affect their toxicity on livestock or humans. Therefore, we pose the following question: Could ECs represent an important and so-far neglected part of ergot toxicity? For that purpose, we quantified ECs content across a large set of ergot sclerotia and present the basic cytotoxicity assays on human cell lines.

## 2. Results

### 2.1. Ergot Alkaloids and Ergochromes Content

**Figure 1:**
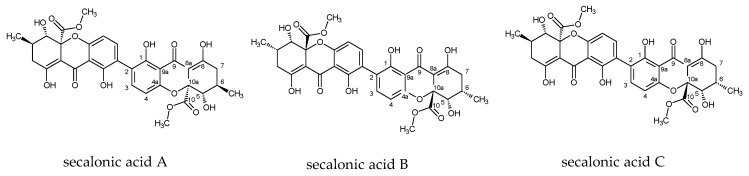
Structures of the main ergochromes from this study, secalonic acid A–C.

**Figure 2:**
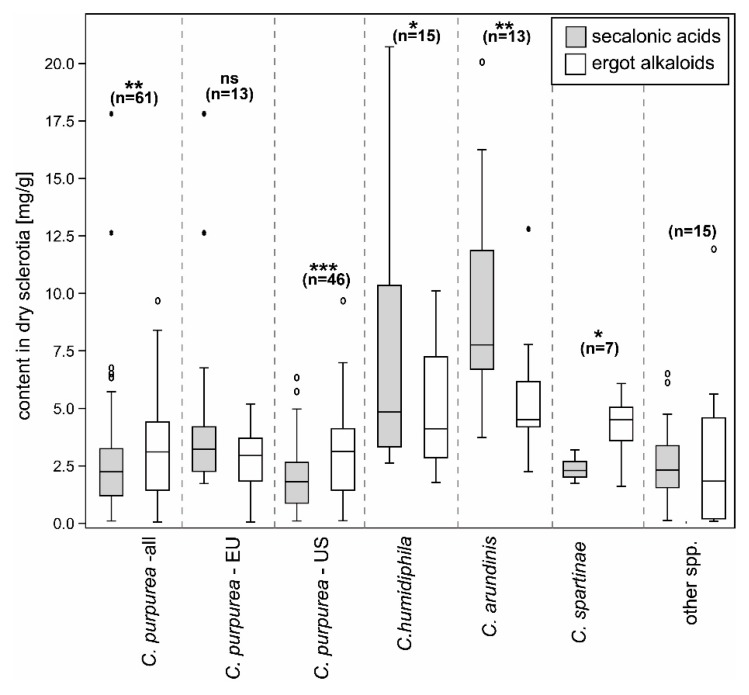
Box plot graph summarizing the total content of secalonic acids and ergot alkaloids in the dry sclerotia. The category of other species includes: *Claviceps* sp. 1, sp. 2, *C. capensis*, *C. cyperi*, *C. fimbristylidis*, *C. macroura*, *C. monticola*, *C. nigricans*, and *C. pazoutovae* (Table S1). *** *p* < 0.001, ** *p* < 0.01, * *p* < 0.05, ns—not significant, in the Wilcoxon Signed Rank test.

**Figure 3:**
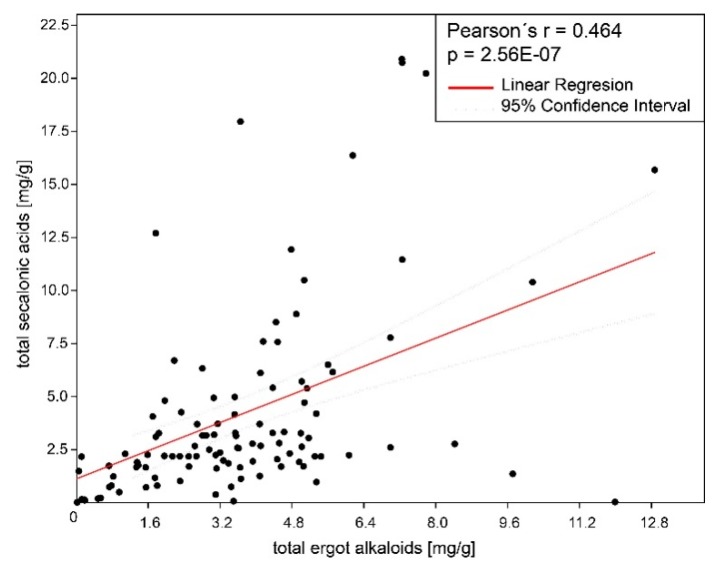
Linear regression of total secalonic acid (SAA, SAB, SAC) and ergot alkaloid content in sclerotia.

**Figure 4:**
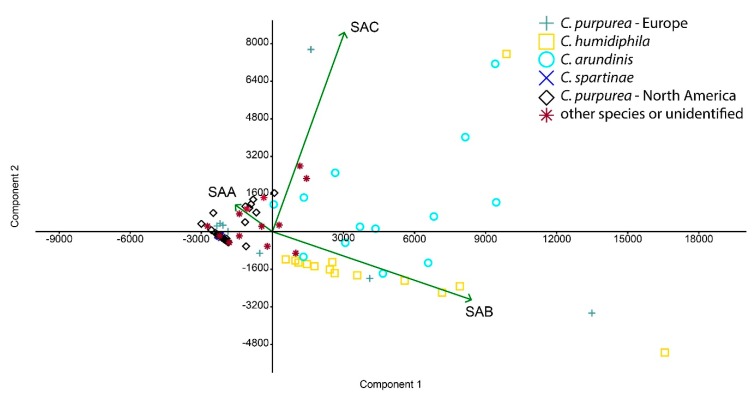
Principal component analysis showing relatedness of samples based on content of SAA, SAB, and SAC. PCA (Principal Component Analysis) axis 1 and 2 explained 76.3% and 16.1%, respectively.

Three major SAA, SAB, and SAC and two minor ECs, endocrocin, and ergochrysin were identified in sclerotia. The average content of all SAs and EAs across all 111 samples was 4.08 mg/g (SD 4.39) and 3.58 mg/g (SD 2.46), respectively (Table 1, Figure 1 and Figure 2, Table S1). These values were statistically not different (paired *t*-test, *p* = 0.3) and moderately, but significantly correlated (Figure 3, Pearsson coefficient 0.46, two tailed *t*-test, *p* < 0.001). The SAs content and proportion differed substantially between species and between European and North American (NA) populations of *C. purpurea s.s. Claviceps arundinis* and *C. humidiphila* contained significantly more SAs than EAs and had the highest content of all SAs among analyzed species. The dominance of SAs over EAs was also found in *C. capensis*, *C. macroura*, *C. monticola*, *C. pazoutovae*, and *C. fimbristylidis* (not tested for significance due to the low sample size). The opposite ratio was found in *C. purpurea* (all sample set), *C. spartinae* (significant different in both cases), *C. cyperi*, and *C. nigricans* (not tested for significance). European *C. purpurea s. s.* population had similar content of SAs and EAs, whereas the NA population had significantly more EAs than SAs (Table 1, Figure 2, Table S1). Both populations differed significantly in SAs (Mann-Whitney test, *p* < 0.005), but not in EAs content. The spectrum of SAs was shown to have an obvious chemotaxonomic value which separates European *C. purpuera* and *C. spartinae* (SAA as a dominant and often single secalonic acid), from the NA *C. purpurea* (SAC dominant, followed by SAB and SAA) and *C. arundinis* (SAB dominant, followed by SAC) and *C. humidiphila* (SAB dominant, SAC minor) (Figure 4, Table S1).

### 2.2. Biological Activity In Vitro

**Figure 5:**
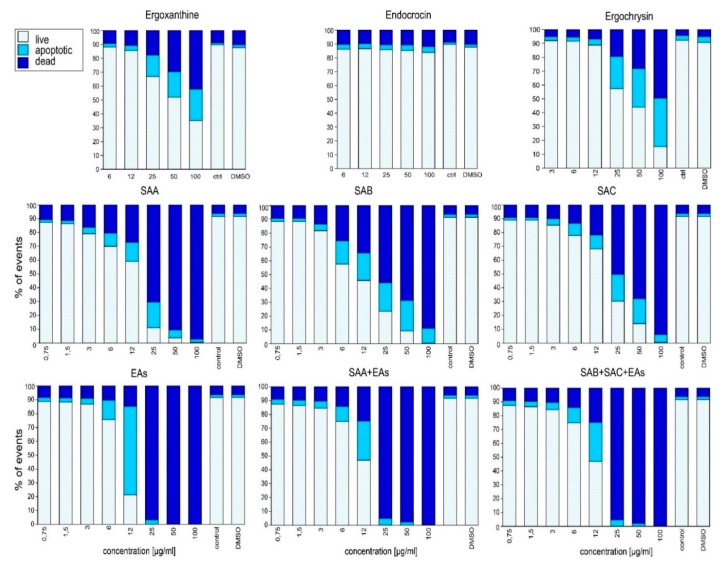
Live, Apoptotic, and dead cell events in Jurkat cells after 24 h incubation. Representative toxicity values for endocrocin, ergochromes, ergotamine, and the combinations of SAs and ergotamine. Combinations reflect the mixtures of SAs and EAs found in the real sclerotia. Cells are shown as events detected by flow cytometry and expressed in percentages (total amount of cells = 100% of events).

**Figure 6:**
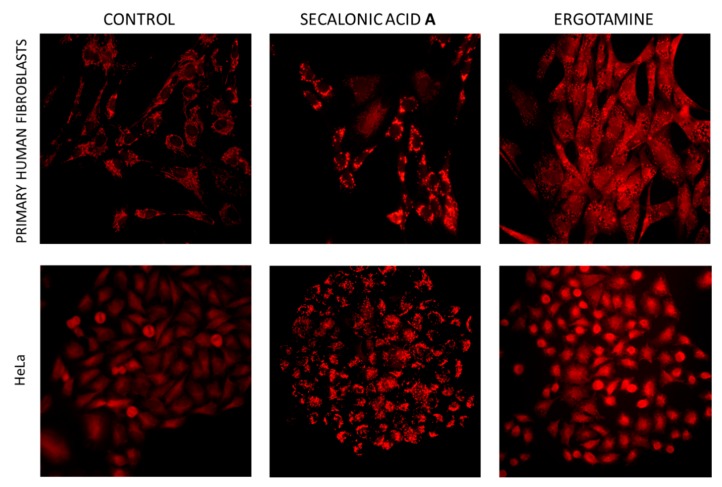
Mitochondrial architecture and Mitotracker positivity in HeLa cells and primary skin-derived fibroblasts. Cell cultures grown on glass cover slips were treated for 24 h with 12 μg/mL secalonic acid A or ergotamine and in vivo incubated with MitoTracker^®^ Red CMXRos. Magnification 20×.

Studied compounds were tested using cancer-derived Jurkat (Figure 5) and HeLa cell lines along with primary skin-derived fibroblasts (Figure 6). Toxicity tests on Jurkat cells (24 h exposure, Figure 5) showed that endocrocin (anthraquinone) did not cause apoptosis or cell death even in extremely high concentrations (LD50 705.5 μg/mL). The toxicity of ergoxanthine (LD50 142.0 μg/mL) and ergochrysin (LD50 118.8 μg/mL) was lower in comparison to SAs and EAs. Toxicity after 24 h exposure to SAA, SAB, SAC, and EA began at the lowest tested concentration (0.75 μg/mL), with 12 (EAs) and 25 (SAs) μg/mL resulting in 50%, and 50 (EAs) and 100 (SAs) μg/mL resulting in 100% of dead or apoptotic cells in all variants. The fraction of apoptotic and dead cells only slightly differed between these compounds, with EAs (LD50 13.5 μg/mL) showing the highest toxicity, followed by SAA (LD50 19.8 μg/mL), SAB (LD50 35.9 μg/mL), and SAC (LD50 36.5 μg/mL). For SAA + EAs (LD50 12.8 μg/mL) and SAB + SAC + EAs (LD50 15.4 μg/mL) neutral to synergistic effects on the number of dead cells could be observed. 

Strong effects including changes in morphology and cell contact was observed in human primary fibroblasts and HeLa cells. At concentrations above 25 μg/mL cells incubated with SAA, SAB, SAC, and EA stopped dividing and showed loose stress fibers which started to detach. Striking effects on cellular morphology could be detected using Mitotracker probe (Figure 6). In the case of secalonic acid, concentrations above 12 μg/mL elicited rounded mitochondria with extremely high positivity in greater than 50% of fibroblasts and 100% of HeLa cells, indicating modulation of mitochondrial function, namely proton gradient value. In contrast, specific effects of ergotamine to both HeLa (100% affected cells above 50 μg/mL) and fibroblasts (25 μg/mL) caused the formation of swollen vacuolar structures. We can estimate that these swollen structures (not acidic—negative for Lysotracker probe) could stand for more than half of the cellular volume. Representative data for SAA and ergotamine treatments (various concentrations and simultaneous detection of actin cytoskeleton, mitochondria, and nuclei) are shown as Figure S1.

## 3. Discussion

### 3.1. Ergochrome Quantity and Distribution Across the Species

Ergochrome production in ergot fungi seems to be limited to the section *Claviceps* [1]. This section, represented by *C. purpurea s. l.*, is the most widely distributed section of the genus *Claviceps* and infects the largest number of host plants [1,2]. Due to its cosmopolitan distribution with over 400 potential hosts, including many economically important crops such as rye, wheat, triticale, and barley, the EFSA has selected *C. purpurea s. l.* to be the focus of chemical analysis for food safety concerns [7]. Among the members of *C. purpurea s. l.*, only *C. purpurea s. s.* and *C. humidiphila* infect cereals or cultivated and wild forage grasses and thus have an agricultural importance. *Claviceps cyperi*, infecting *Cyperus* spp. is distributed only in South Africa and is a known causal agent of ergotism in cattle [31]. *Claviceps arundinis* (mostly on *Phragmites*, *Mollinia*, or *Leymus arenarius*) and *C. spartinae* (*Spartina*, *Distichlis*) can be very common in their particular habitats, but their grass hosts are not significantly grazed by animals [2,3,32,33]. Populations of others, i.e., Paleartic *C. nigricans* (*Eleocharis, Scirpus*) or South African *Claviceps capensis* (*Ehrharta*), *C. fimbristylidis* (*Fimbristylis*), *C*. *pazoutovae* (*Ehrharta* and *Stipa*), *C. macroura* (*Cenchrus*), or *C. monticola* (*Brachypodium*) are rare in abundance and their hosts have low palatability [34,35].

Three major combinations of SAs in sclerotium were found and their mutual ratios have taxonomic value. Franck [36] analyzed three *C. purpurea* sclerotia from rye and found SAA as the dominant SA, followed by SAB and SAC, which is concordant with our data for SAs in European *C. purpurea s. s.* Concerning the total content of ECs, the only reliable publication reports 5 mg/g [20] which fully corresponds with our results. Besides SAs, other pigments were also found in several samples (*C. arundinis*, *C. humidiphila*, and *C. spartinae*) but in negligible quantity. The observed quantity of endocrocin (maximum 205 μg/g) corresponds with Franck [20] which reports maximal values of 40 μg/g (Table 1, Table S1).

Based on our data, the total EAs and SAs content in sclerotia was significantly correlated and the amount of toxic compounds in a single sclerotium is thus cumulative. This correlation also shows that measuring EAs can be used to some extent as a proxy for SAs abundance. It is already known that the pigment content of sclerotia is proportional to its alkaloid content and thus a pigments quantification (namely clavorubrin) can provide a method for measuring the samples’ toxicity [37,38].

Both toxin groups are metabolically independent, but their production in sclerotia is contemporary and seems to be regulated by the same stimuli (phosphate level) [39], which can explain the observed correlation. Fungal pigments typically have a light protection role, and this ability is also expected in the case of ECs [39]. Contrary to this, EAs are light sensitive [40] and the correlation between the contents of EAs and SAs could also just be a consequence of light induced degradation of EAs in the sclerotia with primary lower contents of pigments (SAs).

### 3.2. Ecological Role of SAs

From an ecological point of view, SAs can contribute to ergot toxicity (see below) and are thus involved in the protective mutualism known in *C. purpurea s. l.* [41]. SAs can play an important role in the protection of the sclerotia against light or in antibiosis (resistance to microbial attacks). In particular, SAB showed activity against *Bacillus megaterium*, *Escherichia coli*, and *Microbotryum violaceum* [42], and SAA showed antibiotic activity against *Bacillus subtilis*, *Piricularia oryzae* [27], *Microccocus luteus* (MIC 4–8 μg/mL), and *Enterococcus faecalis* (MIC 32 μg/mL) [43]. SAA is also a highly potent non-host specific phytotoxine acting as a possible virulence factor of *Pyrenophora terrestris* [28]. Interestingly, the whole section *Claviceps*, which is unique due to the presence of ECs, also has an extraordinary broad host range [1]. This suggests that ECs could potentially play some role in the virulence cycle, but are surely not essential for it, as was shown in infections test with mutant *C. purpurea* strains with blocked ECs synthesis [39]. 

### 3.3. Ergochromes Toxicity and Significance

The main aim of this study was to determine the quantity of ECs produced by *C. purpurea* and other members of the section *Claviceps* to help assess their potential role in ergotism pathogenicity. The effort to control the toxicosis of forage and human food has generally been successful; however, there has been a resurgence of research interested in the toxicosis of livestock due to increased ergot abundance in recent years [44] and continual reports of ergotism on livestock. 

While many researchers continue to focus on compounds that possess the tetracyclic ergoline ring of ergot alkaloids, we showed that so far neglected SAs are equally abundant as alkaloids and have similar toxicity to cell cultures. While our data shows the essential first step, further research into the toxicity of SAs is needed to elucidate their real impact on human and animal health. Understanding the connection of SAs to ergotism might help explain some of the unexplained aspects of egotism. Klotz [9] published a thorough review of the inconsistent nature and occurrence of ergotism in livestock. While his review only covered the toxicosis of *Claviceps*-derived ergotamine and ergocristine and *Epichloë*-derived ergovaline, he was still able to determine that the impacts of alkaloids on livestock are not caused by the sole action of a single toxin but rather the combined impact and synergistic action of multiple alkaloids. This was evident as many researchers were generally unsuccessful in replicating the complete effects of ergotism by introducing individual or even combinations of multiple alkaloids to livestock. For example, individual applications of ergovaline and ergotamine and combined applications of ergocornine, ergocryptine, and ergocristine were unable to produce gangrenous ergotism or fescue foot in all of the exposed livestock [45,46,47,48]. Similar inconclusive results were also observed for other aspects of ergotism such as fat necrosis and male-specific effects [9]. Such inconsistencies might be the results of concentration levels, alkaloid proportions, isomeric forms, accumulation, as well as individual’s genetic predispositions, prior exposures, and ambient environments.

Therefore, SAs might represent a missing piece in the larger picture of ergotism on livestock. A few experiments on living animals were conducted with SAs. The major compound of ergot sclerotia, SAA, is lethal to mice at the peritoneal injected doses 50–100 mg/kg [27]. In another study, SAA caused edema and inflammation in rats after the intraperitoneal application of 12.5–50 mg/kg [49]. This toxicity is comparable to well recognized toxin SAD which LD50 for mice in intraperitoneal injection was reported as 42 mg/kg [50] or in a range of 26.5–51.7 mg/kg [24]. Teratogenic effects were observed in rats injected with SAD and teratogenic activity started at doses of 5 mg/kg body weight [25,51]. Data from humans are missing, but it is known that SAD produces cleft palate as the only malformation in fetal mice which is of potential relevance to human health [52]. Furthermore, SAs have various biological effects at non-toxic doses. SAA at the concentrations 0.15–0.75 mg/kg injected peritoneally to mice affect the metabolism of dopaminergic neurons [53,54].

In cases of cell line cultures, the toxicity to cancer cells is much higher than for normal cells. SAA is toxic against cultured mouse (IC50 = 0.5 μg/mL, [55]) or human leukemia cells (ED50 = 3.5 μg/mL, [43]). These values roughly correspond to the LD50 ranging between 20–37 μg/mL found in SAA-SAC for Jurkat cells in our study. The IC50 for murine melanoma cells was 1.8 μg/mL for SAA and 0.18 μg/mL for SAD, but to affect healthy keratinocytes a 3.5× (SAA) or 13× (SAD) higher concentration was needed [56]. SAD is also cytotoxic against various carcinoma cell lines at very low concentrations (IC50 = 0.05–0.76 ug/mL) and its mechanism of function is the best studied among all ECs [57].

Thus, the question is whether SAs of ergot origin can negatively affect animal health. The only available data are for SAD, and optical antipode of SAA, which have very similar toxicity to animal models and potentially have the same mode of action [58]. Whereas, at the intraperitoneal application, the LD50 for SAD and SAA ranges between 25–37 mg/kg (applied one times) while there is an 11-fold lower sensitivity when introduced by an oral route in mice [59]. Thus, an LD50 of around 400 mg/kg body weight can be close to toxicity values found in field conditions. Scenarios accounting for repeated applications of sublethal doses increase the toxicity by approximately three times as toxic effects of SAD were found to be cumulative in a five-day feeding experiment, which indicates that the toxin can be accumulated within the organism [59]. The question, what is the exposition of livestock to SAs under natural conditions, remains unanswered. In EAs the lowest save doses (no-observed-adverse-effect levels) are 0.22–0.60 mg/kg body weight per day [7]. Concerning the fact that SAs and EAs have very similar concentrations in ergot, we can expect that animals encounter similar doses. Based on our above review, the lethal toxicity of SAs is more than 125 times lower than in EAs and their importance in the acute intoxication can play a role in case of highly contaminated fodder only. Nevertheless, SAs have various biological activities in much lower doses (i.e., 0.15 mg/kg injected peritoneally, [53,54]) corresponding to expositions in natural conditions. 

### 3.4. Mode of Action

SAs exhibit various bioactivities, generally cytotoxic and cytostatic. SAD and SAF exhibit antitumor activity in low micromolar ranges, including induction of cell cycle arrest in G1 or induction of apoptosis [60,61]. The molecular mechanism behind the antitumor selectivity and toxicity remain putative. Opposite to cytotoxic effects, SAA applications in a Parkinson’s disease mouse model protected against dopaminergic neuron death [53]. The best studied SA, SAD, exhibited various modes of cytotoxicity towards multidrug resistance cells due to induction of ABCG2 degradation via calpain-1 activation [57]. Our observations agree with this general view that particular bioactivities are cell type dependent (in our case leukemic cell line, adenocarcinoma, and primary human fibroblasts) in terms of effective concentrations and particular phenotypes (proliferative block, loss of stress fibres, proportion of apoptotic cells). The hallmark physiological and morphological changes in all cell types treated with SAs was transformation of the typical mitochondrial network to isolated rounded mitochondria with extremely high positivity for Mitotracker probe, indicating interference with mitochondrial function. For example, electron-transport chain functions and proton gradient levels. This is in agreement with previously published results that SAD uncoupled the oxidative phosphorylation in isolated rat mitochondria [62]. Another indication, that mitochondria could be the cellular target for SAs is based on the observation that SAA protects dopaminergic neurons from 1-methyl-4-phenylpyridinium induced cell death via the mitochondrial apoptotic pathway [54]. 

## 4. Conclusions

Despite the fact that ergochromes do not play a significant role in acute toxicity of ergot, the complexity of the secondary metabolites produced by *Claviceps* species including secalonic acids points to a more complex fungus-grazing animal interaction. Grazing animals consuming ergotised grasses are constantly exposed to doses of secondary metabolite mixtures that can have a profound biological effect on their physiology or development. This publication is focused on the major secondary metabolite family, SAs, which can offer the tempting explanation to the complex ecological interaction between the herbivore and the fungus. Historically, the major body of research was focused on the ergot alkaloids. The observation that *Claviceps* secondary metabolome is much more diverse than anticipated, providing new research direction. Future research needs to examine metabolism, absorption, and excretion of SAs to determine their potential in short term versus long term toxicosis. For example, the described effect of SAs on mitochondria (temping molecular mechanism behind the bioactivity) indicating high interference with function needs detailed studies to be understood completely. These research directions are essential to understand ergotism itself in its complexity and to help determine whether forage and human food contaminated with *Claviceps purpurea* should be monitored for the control of ECs content, as proposed in this publication.

## 5. Materials and Methods

### 5.1. Specimens Analyzed

The analyzed sclerotia (*n* = 111) covers material from four continents. All 13 agriculturally and environmentally important ergot species producing ergochromes, were collected from the wild as well as cultivated grasses and cereals; i.e., *C. purpurea s.s*. (collections from Europe, North America, New Zealand), *C. humidiphila* (Europe), *C. arundinis* (Europe), *C. nigricans* (Europe), *C. spartinae* (Europe), *C. cyperi*, *C. fimbristylidis*, *C. macroura*, *C. monticola*, *C. pazoutovae*, (all from South Africa), and two undescribed *Claviceps* sp. (USA, New Zealand). Materials originated from previous studies [2,34,63] or were collected during the course of this study (Table 1, Table S1). Sclerotia were identified using the ITS rDNA sequence barcode. DNA from sclerotia was isolated using the fast NaOH protocol [64] for European samples or using the PowerPlant Pro DNA Isolation Kit (MoBio/Qiagen) for US/NZ samples. PCR and sequencing of the ITS barcode was performed according to Pažoutova et al. [2]. DNA and chemical analyses were done from the same sclerotium in the case of larger sclerotia. In cases of very small sclerotia, chemical analysis was performed from the whole sclerotium and DNA based identification was completed using a sclerotium collected from the same or adjacent grass spiclet. 

### 5.2. Sample Preparation, Extraction, and HPLC Analyses

Pulverized sclerotia (1–10 mg) were mixed with extraction mixture dichloromethane and concentrated ammonia (500:1, *v*:*v*, 0.5–2.0 mL) and gently stirred for 1 h. Supernatant was separated by centrifugation and kept in the freezer until use. The same HPLC instrumentation and method as published earlier [1] was used for the analysis of EAs and ECs in sclerotia of all *C. purpurea s.s.* analyzed.

### 5.3. Ergot Alkaloids and Ergochromes Identification and Quantification

#### 5.3.1. General Workflow

Identification of EAs was based on retention time which was compared with standard compounds and UV–VIS spectra of individual compounds. Secalonic acids A, B, and C, were isolated and identified using UV–VIS spectra, FTMS, NMR, and optical rotation. The isolated compounds were used as chromatographic standards for quantification of their content in individual sclerotia. Further isolated ECs were determined as endocrocin and ergochrysin by the same procedures as used for the SAs. In both cases the data obtained were in agreement with previously published data [20].

#### 5.3.2. FTMS

Samples were measured using 15T solariX FTMS equipped with an ESI/MALDI ion source and ParaCell (Bruker Daltonics, Billerica MA). The analysis was performed using an electrospray ionization (ESI) in a positive ion mode as described in Flieger et al. [65] with the following differences: The collision energy was kept at −15.5 V, the mass range for MS data acquisition started at *m*/*z* 150 a.m.u., resulting in a resolution of 250,000 at *m*/*z* 400. The detailed FTMS data for SAA-SAC are presented in Figure S2.

#### 5.3.3. NMR

NMR spectra were measured on a Bruker Avance III 600 MHz spectrometer (600.23 MHz for 1H, 150.93 MHz for ^13^C) in CD3CN (20 and 30 °C). Residual signals of solvent were used as an internal standard (at 20 °C: δ_H_ 1.941, δC 1.41, at 30 °C: δ_H_ 1.936, δ_C_ 1.35). NMR experiments: ^1^H NMR, ^13^C NMR, COSY, ^1^H-^13^C HSQC, ^1^H-^13^C HMBC, and ROESY were performed as described in Stodůlková et al. [66]. Detailed NMR data of the identified compounds are provided in Table S2 (^13^C NMR) and Table S3 (^1^H NMR).

#### 5.3.4. Quantification of Ergot Alkaloids and Ergochromes

A standard solution of quantified compounds, i.e., ergotamine and ergochrysin, were prepared in methanol and SAA in acetone at final concentrations of 31.75, 62.5, 125, 250, 500, and 1000 μg mL^−1^. The calibration graphs were constructed by plotting the integrated peak areas of individual compounds versus concentration. The following linear regression equations and correlation coefficients were obtained: Ergotamine; y = 5298.3x, *R*^2^ = 0.9994, UV = 315nm; secalonic acid A; y = 14537x + 6452, *R*^2^ = 0.9995, UV = 315nm; ergochrysin; y = 6205.7x − 2015, *R*^2^ = 0.9991, UV = 315nm.

### 5.4. Biological Activity Testing

For toxicity studies, immortalized T-lymphocyte-derived cancer cell line Jurkat was cultivated in 96-well plates at a density of 2 × 10^5^ cells in a final volume of 300 μL of RPMI1640 (LONZA, USA). Cells were treated with different concentrations of SAs, ergoxanthin, endocrocin, ergotamine, and a combination of SAA and ergotamine (1:1) and SAB, SAC, and ergotamine (0.5:0.5:1) (Figure 5). Cells cultured in RPMI1640 and in RPMI1640 with only DMSO were only used as negative controls. After the incubation (24 h), cells were washed in PBS containing 0.02% gelatine and 0.01% sodium azide (Sigma-Aldrich, St. Louis, MO, USA). Hoechst 33258-stained cells were analyzed with the FACS LSRII instrument (BD Biosciences, San Jose, CA, USA) and FlowJo 10.5.3 software (Tree Star, Ashland, OR, USA).

For fluorescent microscopy adenocarcinoma cell line HeLa and primary human skin-derived fibroblasts were used. Cells were cultivated in DMEM medium with 10% FCS (Gibco, Invitrogen, Grand Island, NY, USA) and seeded on glass cover slips (up to density 50%) in 24-well plates. Cells treated with different concentrations of tested compounds were cultured in DMEM, wells supplemented with only DMSO were used as negative controls. After 24 h incubation, cells were incubated (10 min, Lysotracker^®^ Red, or MitoTracker^®^ Red CMXRos (Molecular Probes-Invitrogen, Carlsbad, CA, USA) and fixed (3.7% paraformaldehyde in PBS, 20 min, RT), permeabilized (0.1% Triton X-100 in PBS), blocked (1% BSA in PBS), and stained with Phalloidin-Alexa Fluor^®^488. All fluorescent dyes were from Molecular Probes (Invitrogen Carlsbad, CA, USA). Morphological observations were performed using Olympus IX71microscope equipped with DP70 camera 20× objective. Nuclei were stained and specimens mounted using Fluoroshield DAPI (Sigma Aldrich).

### 5.5. Statistical Analysis

Data were visualized on PCA (Principal Component Analysis). The normality of the data was tested using Chi-squared test and the correlation between SAs and EAs production was done using the linear Pearson test. A non-parametric Wilcoxon signed rank test was used to test the null hypothesis of no difference in the EAs and ECs concentrations within the particular species or population. Non parametric Mann-Whitney test or parametric paired *t*-test was used to compare EAs and SAs content between populations or across all samples. These statistical analyses were done using the PAST 3.25 software [66]. The LC_50_ values of tested compounds were calculated using probit analysis [67,68] using Microsoft Excell^®^ Professional Plus 2013 software (Microsoft Corp., Redmond, WA, USA).

## Figures and Tables

**Table 1:** Concentration summary of ergochromes and ergot alkaloids in *Claviceps* spp. sclerotia. Content in dry sclerotia is expressed as minimum-average (standard deviation)-maximum. The category of other species includes: *Claviceps* sp. 1, sp. 2, *C. capensis*, *C. cyperi*, *C. fimbristylidis*, *C. macroura*, *C. monticola*, *C. nigricans*, and *C. pazoutovae* (Table S1).

Taxon	# Samples	Total Secalonic Acids (mg/g)	Endocrocin, Ergochrysin (µg/g)	EAs (mg/g)
*C. purpurea*—Europe	13	1.66–5.05 (4.87)–17.96	0.00–17.51 (29.64)–97.58	0.00–2.69 (1.65)–5.17
*C. purpurea*—NA	46	0.02–1.91 (1.43)–6.33	0.00–3.13 (13.75)–84.82	0.02–2.96 (1.99)–9.71
*C. purpurea*—all	61	0.02–2.59 (2.81)–17.96	0.00–6.09 (18.96)–97.58	0.00–3.03 (2.03)–9.71
*C. humidiphila*	15	2.56–7.54 (6.04)–20.90	0.00–14.96 (16.07)–50.31	1.70–4.77 (2.38)–10.16
*C. arundinis*	13	3.70–9.67 (5.08)–20.23	0.00–50.28 (34.18)–126.93	2.18–5.32 (2.77)–12.87
*C. spartinae*	7	1.67–2.29 (0.49)–3.15	0.00–56.84 (68.65)–205.13	1.55–4.20 (1.42)–6.07
other spp.	15	0.03–2.66 (1.93)–6.50	0.00	0.00–1.77 (3.20)–11.99
all samples	111	0.02–4.08 (4.39)–20.90	0.00–9.01 (24.55)–205.13	0.00–3.57 (2.46)–12.87

## Supplementary Materials

The following are available online at https://www.mdpi.com/2072-6651/11/8/439/s1, Table S1: List of analyzed ergot samples and the content of ergochromes and ergot alkaloids, Table S2: ^13^C NMR data for secalonic acid A–C, Table S3: ^1^H NMR data for secalonic acid A–C, Figure S1: Cell cultures grown on glass cover slips were treated for 24 h with secalonic acid A or ergotamine and in vivo incubated with MitoTracker^®^ Red CMXRos, fixed, permeabilized, and labeled with Phalloidin-Alexa Fluor^®^488, Figure S2: FTMS data for secalonic acid A–C.
