# Supplementary material for: Ergochromes: Heretofore Neglected Side of Ergot Toxicity
Source: Toxins (Basel). 2019 Jul 25;11(8):439. doi: 10.3390/toxins11080439 (PMC6722540; doi:10.3390/toxins11080439)
Supplement: Supplementary file 1 [file toxins-11-00439-s001.zip › toxins-545211.pdf]

Supplementary Materials: Ergochromes: Heretofore Neglected Side of Ergot Toxicity

Miroslav Flieger, Eva Stodůlková, Stephen A. Wyka, Jan Černý, Valéria Grobárová, Kamila Píchová, Petr Novák, Petr Man, Marek Kuzma, Ladislav Cvak, Kirk D. Broders and Miroslav Kolařík

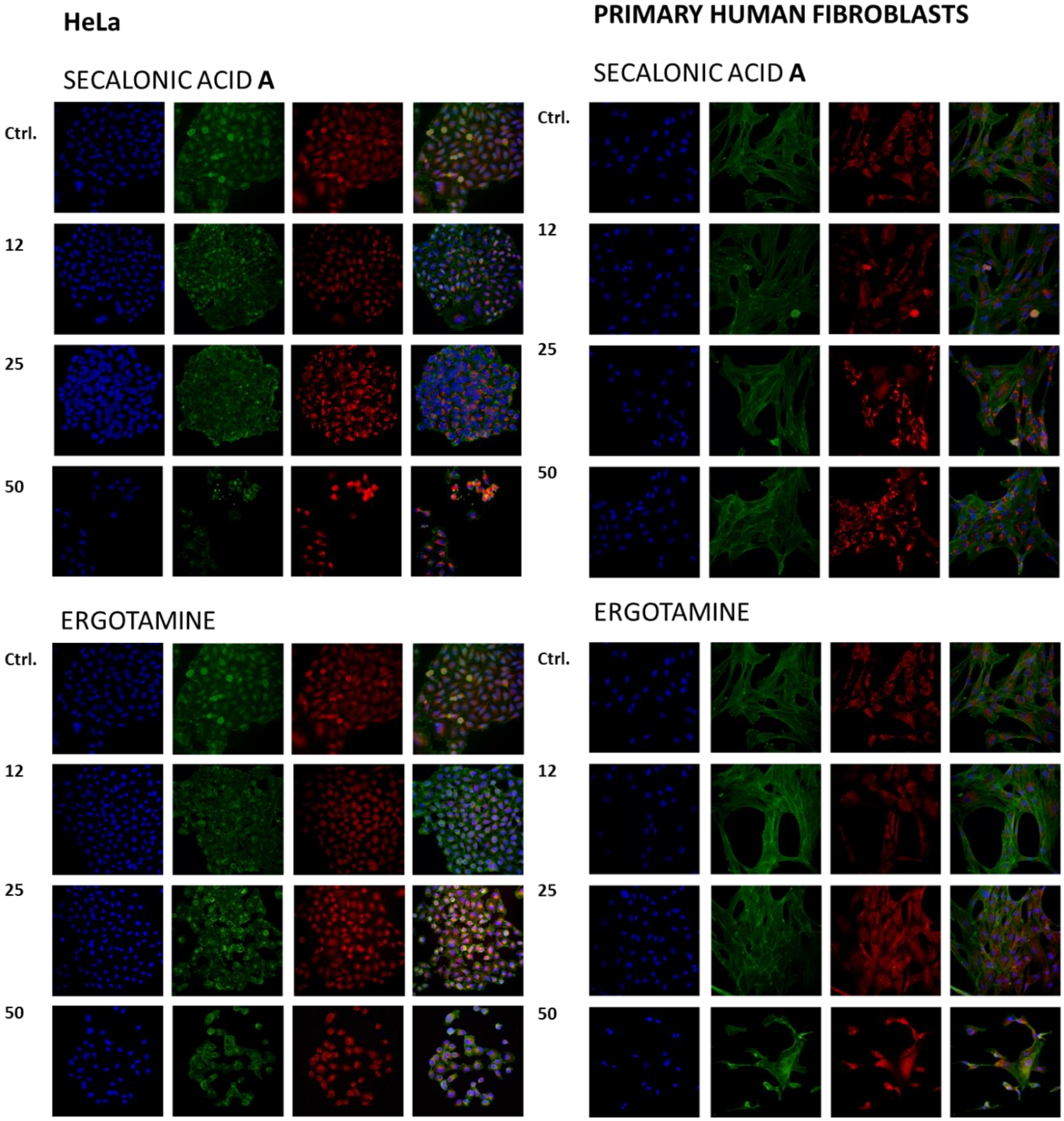

Figure S1. Cell cultures grown on glass coverslips were treated for 24 h with secalonic acid A or ergotamine and in vivo incubated with MitoTracker® Red CMXRos, fixed, permeabilized and labelled with Phalloidin-Alexa Fluor®488. Magnification 20x.

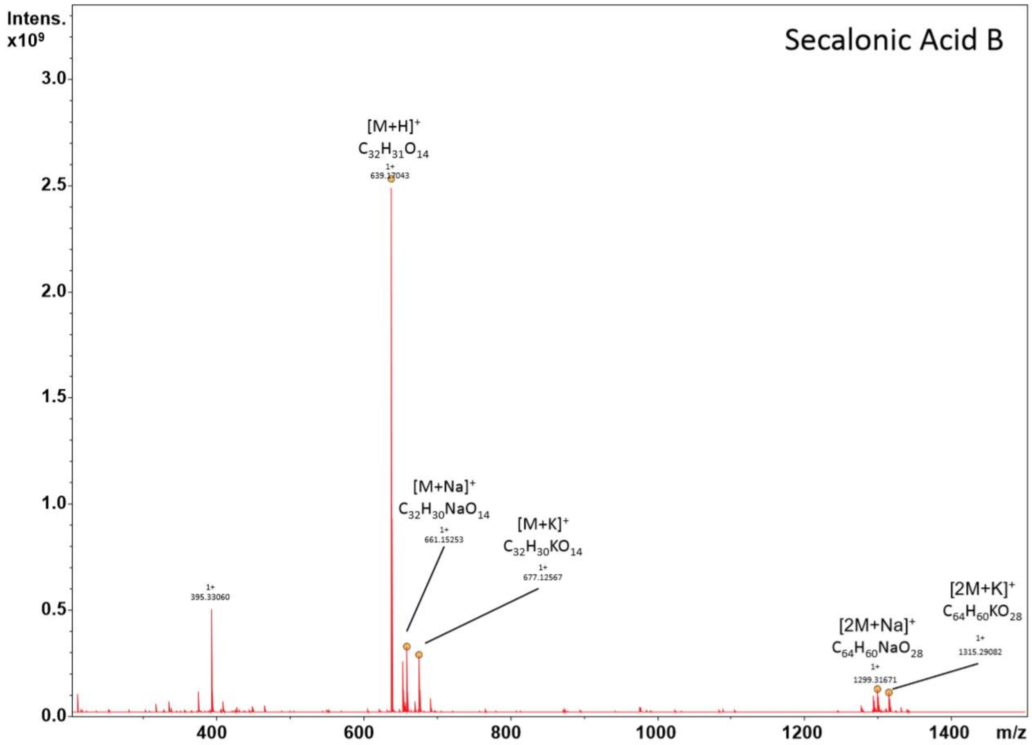
Intens. x10⁹
Secalonic Acid B
[M+H]+
$C_{32}H_{31}O_{14}$
639.17043
[M+Na]+
$C_{32}H_{30}NaO_{14}$
661.15253
[M+K]+
$C_{32}H_{30}KO_{14}$
677.12567
395.33060
[2M+Na]+
$C_{64}H_{60}NaO_{28}$
1299.31671
[2M+K]+
$C_{64}H_{60}KO_{28}$
1315.29082
m/z

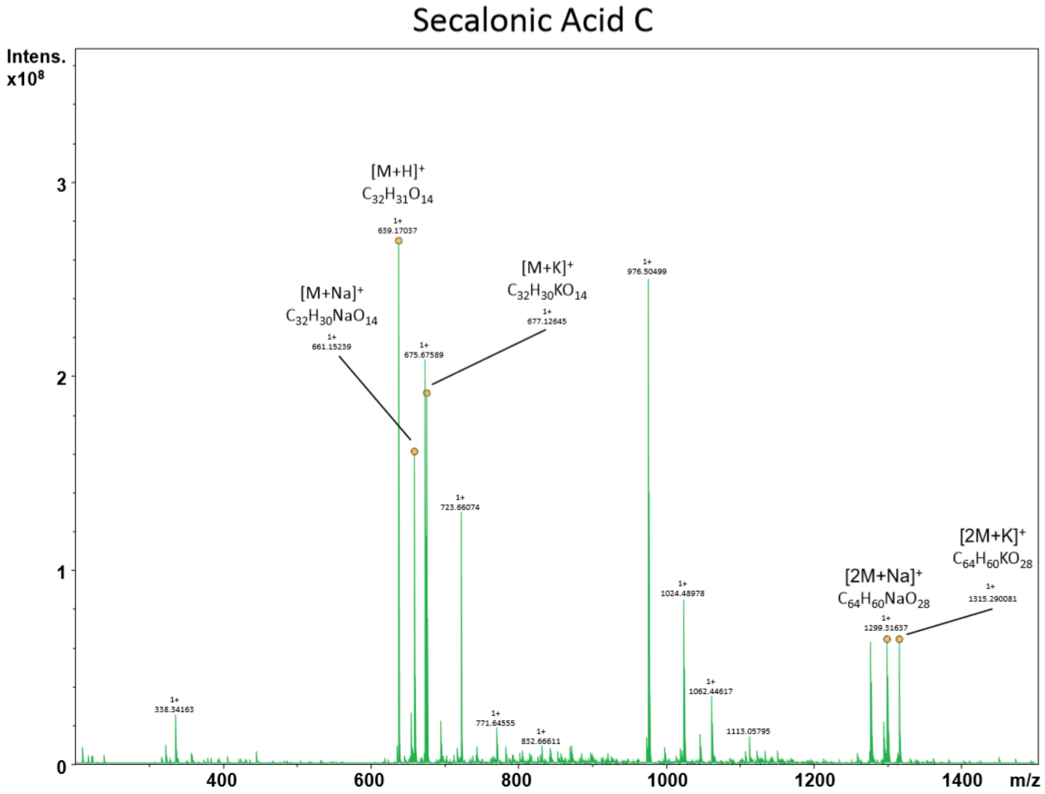
Secalonic Acid C
Intens. x10⁸
[M+H]+
$C_{32}H_{31}O_{14}$
639.17037
[M+Na]+
$C_{32}H_{30}NaO_{14}$
661.15239
675.67589
[M+K]+
$C_{32}H_{30}KO_{14}$
677.12645
976.50499
723.66074
1024.48978
1062.44617
1113.05795
338.34163
771.64555
832.66611
[2M+Na]+
$C_{64}H_{60}NaO_{28}$
1299.31637
[2M+K]+
$C_{64}H_{60}KO_{28}$
1315.290081
m/z

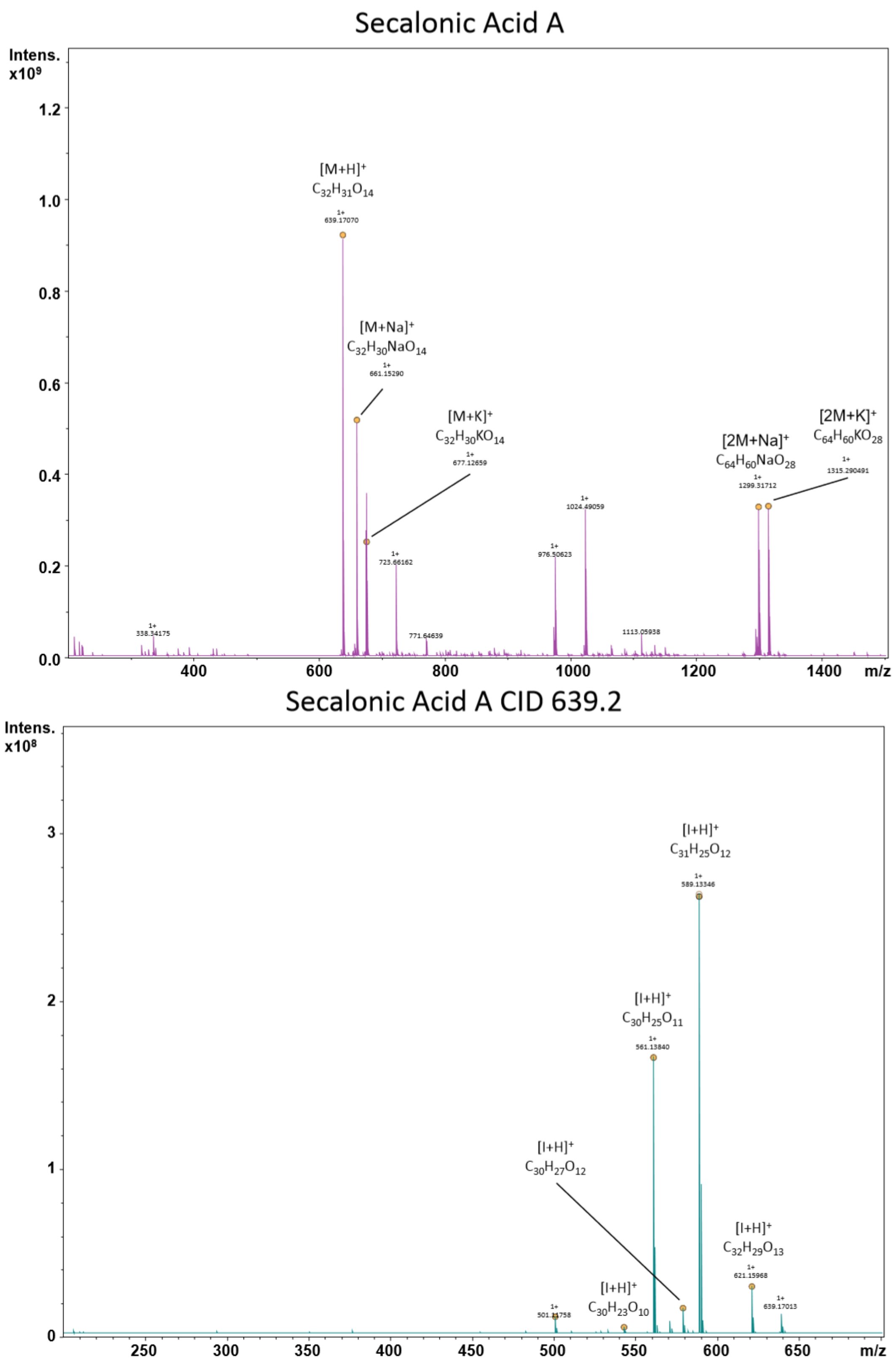

Figure 2. FTMS data for Secalonic Acid A–C.

Table S2. NMR data for Secalonic acid A–C.

Atom	SA A		SA B		SA C	
**Number**	**δC**	**m**	**δC**	**m**	**δC**	**mC**
**1**	158.44	s	160.32	s	160.89	s
**2**	117.21	s	119.32	s	119.96	s
**3**	140.13	d	141.05	d	141.66	d
**4**	107.39	d	108.55	d	109.14	d
**4a**	158.81	s	158.75	s	159.31	s
**5**	75.13	d	71.81	d	72.44	d
**6**	29.80	d	29.50	d	30.11	d
**7**	35.72	t	33.46	t	34.07	t
**8**	178.11	s	181.54	s	182.10	s
**8a**	101.61	s	101.48	s	102.07	s
**9**	186.47	s	189.00	s	189.59	s
**9a**	106.21	s	107.59	s	108.23	s
**10a**	85.07	s	86.02	s	86.63	s
**6-Me**	17.65	q	17.76	q	18.33	q
**10a-CO**	169.92	s	172.25	s	172.83	s
**10a-OMe**	52.72	q	54.12	q	54.66	q
**1'**					160.87	s
**2'**					119.28	s
**3'**					141.88	d
**4'**					109.16	d
**4'a**					160.82	s
**5'**					77.82	d
**6'**					31.36	d
**7'**					37.45	t
**8'**					180.07	s
**8a'**					103.52	s
**9'**					189.15	s
**9'a**					108.40	s
**10'a**					86.74	s
**6'-Me**					18.80	q
**10'a-CO**					171.71	s
**10'a-OMe**					54.10	q

m – multiplicity

Table S3. ^{1}H NMR data for Secalonic acid A–C.

Atom	SA A			SA B			SA C		
**Number**	**δH**	**mH**	***J* [Hz]**	**δH**	**mH**	***J* [Hz]**	**δH**	**m**	***J* [Hz]**
**3**	7.456	d	8.5	7.431	dd	0.4, 8.4	7.416	d	8.5
**4**	6.626	d	8.5	6.553	d	8.4	6.538	d	8.5
**5**	3.812	dd	5.8, 11.1	4.068	dd	1.7, 3.2	4.071	d	1.5
**6**	2.309	dddq	6.2, 6.5, 10.9, 11.1	2.085	m	-	2.083	m	-
**7**	2.660	dd	6.2, 19.3	2.414	m	-	2.410	m	-
	2.471	dd	10.9, 19.3	-	-	-	-		
**1-OH**	11.602	s	-	11.728	d	0.4	11.720	s	-
**5-OH**	6.001	d	5.8	3.690	dd	1.4, 3.2	n.d.		
**6-Me**	1.038	d	6.5	1.090	d	6.8	1.090	d	6.6
**8-OH**	13.601 T	br s	-	13.908	s	-	13.906 T	br s	-
**10a-OMe**	3.607	s	-	3.666	s	-	3.661	s	-
**3'**							7.440	d	8.4
**4'**							6.595	d	8.4
**5'**							3.854	d	10.9
**6'**							2.410	m	-
**7'**							2.687	dd	5.6, 18.6
							2.359	m	10.8, 18.6
**1'-OH**							11.582	s	-
**5′-OH**							n.d.		
**6'-Me**							1.090	d	6.6
**8′-OH**							13.680 T	br s	-
**10'a-OMe**							3.634	s	-

T—tentative assignment, n.d. - not detected, m - multiplicity
